# Progress in Medicine

**Published:** 1896-12-05

**Authors:** 


### Progress in Medicine.

### DISEASES OF NERVOUS SYSTEM.

**Amyotrophic Lateral Sclerosis.** — Joseph Collins[1] reports two cases with autopsy. He points out that the disease is much more uncommon than is generally supposed, if we may come to such a conclusion from the scarcity of well-authenticated cases with the pathological findings which have been published, Collins having been able to find but seventy-two such cases in an extended search through the literature. Limitations of space preclude our citing the complete and well illustrated reports; but Collins reminds us that the typical clinical manifestations of the disease have been found associated with changes confined to the anterior horns, and no involvement of the lateral columns[2]; and with degeneration of the entire motor tract, the primary and the secondary neuron.[3] Again, cases have been observed in which anatomical investigation has shown degeneration of the motor and sensory systems. Hektoen has recently reported a case of this kind, a typical case of amyotrophic lateral sclerosis, with bulbar paralysis and a posterior sclerosis. Marie[4] found degeneration in the columns of Goll; Oppenheim[5] found degeneration in Burdach's column; Charcot and Marie[6] have described degeneration in the columns of Goll; while Maeli[7] found degeneration in the column of Burdach. It is thus seen that the morbid changes are not very constant.

The common pathological findings are destruction of cells in the posterior area of the medulla and the anterior cornu of the cord, and a degeneration of the pyramidal tracts; but with these conditions there is considerable variation, particularly in respect to the condition of the anterior nerves of the spinal cord, their terminations at the periphery, and the condition of the cranial nerves. It is in accordance with our knowledge of secondary degeneration and our conception of the anatomical and physiological unit, the neuron, to believe that when the anterior horn cells are diseased that the anterior roots will suffer in proportion and in intensity. But cases[8] of amyotrophic lateral sclerosis have been described in which the anterior horn cells have been diseased, and the anterior root and motor spinal nerves found intact, while in the same case the cranial nerves arising from diseased nuclei showed well-marked degeneration.

Other cases of typical amyotrophic sclerosis clinically have been found associated with the lesions of poliomyelitis anterior chronica,[9] with atrophy of the ganglion-cells in the anterior horns, arterial and venous hyperæmia, multiple hæmorrhages, incipient syringomyelia[10]; not to mention those cases which were shown on autopsy to have been due to internal hydrocephalus and tumour of the cord and cerebellum,[11] nor those of Kahler and of Schultze in which the symptoms of amyotrophic lateral sclerosis were found associated with gliosis. As to whether the disease is a form of progressive muscular atrophy, as is held by Gowers, Leyden, and some others, or whether the primary lesion is a degeneration of the pyramidal tract and secondary involvement of the grey matter, as was suggested by Charcot and believed by many neurologists, is a question that will be settled when a sufficiently large number of cases, with pathological findings, have been recorded.

In conclusion, Collins remarks that the belief that the affection of the grey matter is secondary or deuteropathic in amyotrophic lateral sclerosis is more in keeping with the basic anatomical units of the nervous system, the neuron, than is the view taken by

Leyden and Gowers. We know that the centre for nutrition of the neuron is the cell-body of the neuron, and that parts most remote from the cell-body are the first to show degeneration when the nutrition of the centre is affected. In the cases reported, and particularly in Case I., slowly proceeding vascular degeneration caused first, lack of nutrition in the perivascular lymph-spaces, and, as it is in these spaces that the ramifications of the end-brushes take place, it is easily conceivable that the degeneration will extend from such loci, and gradually creep up the collaterals and axis-cylinder processes until the entire neuron or neurodendron is involved. This can be taken as the procedure for the changes in the principal neuron, *i.e.*, the one extending from the cortex to the anterior horns. The same vascular and perivascular changes acting upon the dendrites of the cell-body of the secondary neuron, and upon the cell-body itself, will have their deleterious results manifested in the terminal arborisations of the neuraxon, viz., in the muscles, and the manifestation of it then will be muscular atrophy. It is not only possible, but very probable, that in many cases of amyotrophic lateral sclerosis in which the lesion of the pyramidal tracts could be traced from the cortex to the periphery, such cases as were enumerated in the beginning of the paper, the degeneration may have had its starting-point in the ganglionic cells of the cortex, which are the beginning of the fibres of the pyramidal tracts, and extended from there downward; but the fact that the degeneration may occur in that way does not invalidate what has been said about the probable way in which the lesion developed in these cases. That is, it cannot be regarded that the former is the only way that the degeneration may take place; for even one thorough observation, which showed that the lesion in the pyramidal tracts did not extend above the pons, would prove the falseness of such a contention. That there have been plenty of such cases everyone must admit. In Case I., the most accurate methods of investigation were used; for instance, the method of Marchi, which reveals with unerring accuracy any degenerating fibres which are present; but, in this case, the reaction of the tissues above the pons to the stain was that of normal fibres.

From the point of view of symptomatology, Collins states there are but two or three points to arrest our attention, and of these by far the most striking and uncommon one was the waves of spasmodic smiling and laughter in the first patient, and the involuntary chewing, quiet "smacking" movements in the second.

*Treatment.*—Dr. Sighicelli[12] passed electric currents through muscles out of which the blood had been pressed by the use of the Esmarch's bandage, and found such ischæmic muscles to be better conductors of heat and electricity. Four patients were treated in this manner with very favourable results, and from his experiments he concludes: That artificial ischæmia produces a more permeable medium for electric currents to pass through, and only a minimal part of electric power is lost; that after loosening the constriction an enormous efflux of blood to the extremity takes place, which is favourable to the nutrition of the affected muscles. That the muscles, that are thus made anæmic, not only become smaller in volume, but are more permeable for electricity. That a certain degree of anæsthesia in the extremity is produced by the artificial anæmia, which is very desirable in certain sensitive patients, especially children, when strong currents are to be used.

**Tic Douloureux.**—Gilles de la Tourette[13] emphasises the importance of recognising that there are at least two distinct forms of the affection. The first variety is usually due to some peripheral irritation, such as severe cold for instance, affecting the terminal expansions of the nerve. It also frequently supervenes in the course of an infective disease. In this form of neuralgia the affection is benign and ephemeral. The diffential diagnosis between this form and the other, which is persistent, even incurable, is based mainly on the clinical features peculiar to each, and not on ætiological considerations, which are vague and indefinite. In the benign ephemeral form, not only is the shooting pain much less severe than in facial neuralgia of the second type, but it appears due to exacerbations of a continuous pain. In tic douloureux the symptoms are always paroxysmal; in the intervals between the attacks there is complete absence of pain, but when it reappears it reaches the maximum intensity at once, and it ceases as abruptly as it began. At each paroxysm of pain the patient presses on the painful spot, and so forcible and oft-repeated is this pressure that in men the beard is destroyed in the region involved. In addition to pain there are secondary or accessory phenomena, varying according to the affected area, which are of vasomotor, secretory, or trophic nature.

Ewart[14] observes that surgery has thrown light upon some important points, and has furnished us with two great data: (1) The pain is bound up with the peripheral sensory function at least as much as with the central function, a temporary interruption in the peripheral conduction by division or resection of nerves causing only temporary cessation of the pain until a full return of the sensory function of the part, whilst permanent interruption of one branch of the nerve does not protect against its subsequent extension, likewise peripheral, along another branch, whether vicariously or by overflow. This extension or transference of the pain might be explained by implication of any one of the three factors common to the whole nerve—the vasomotor nerves, the nervi nervorum, or the Gasserian ganglion—but which of these is implicated we are not sure. (2) Surgery has also shown that the Gasserian ganglion is a bridge, the cutting of which permanently arrests the pain (this would not result were the cause of pain mainly central); and that, whenever an assumption of the great risks involved is justified, we have in this operation, and perhaps in this operation only, an ultimate and effective resort. The foregoing considerations and varied clinical observations suggest that in all cases there is a functional or *constitutional factor*, and that in some of them this is paramount. The strongest argument in point is the existence of a group of cases where a gouty connection is manifest; this, indeed, seems to be the only kind of etiology which has hitherto been strictly identified by the results of medical treatment. From this point of view we may classify cases as: (1) Those obviously gouty. (2) Those in which the gouty association is rendered probable by the family as well as the personal history. (3) Those in which the family history is unknown, but the previous history includes visceral affections analogous to those which we recognise as gouty, and in which the personal aspect also speaks of gout. These three groups probably make up a large proportion of cases. (4) Into the remaining group, obviously provisional, are relegated the cases where there is no evidence of any connection with gout, either in the family or in the personal history, and all cases of unknown ætiology. Cases attributed to syphilis, or to the pressure of bony or other tumours, would form a special group. They are not under consideration in this paper. The frequency of a gouty association has probably been under-estimated, because it is often not an immediate one.

The following features, frequently noticed in sufferers, may be regarded as pointing to those constitutional influences which, though not necessarily leading to gout underlie the gouty state: (1) The healthy and often ruddy complexion such as we associate with sthenic gout. (2) The presence of Heberden's nodules or of tophi in the ears. (3) A previous history of gravel or of stone. (4) A long history, often beginning in childhood, of gastric, intestinal, and hepatic disturbances of a nervous type. (5) The abiding strength of the pulse, which strikes us as no less remarkable than the resistance of the patient to the effects of long-continued pain and imsomnia. (6) The adverse influence of alcohol and of certain forms of diet. (7) The presence of uric acid sand in the urine was noted in several of the cases related. (8) It is also significant that in two of the male cases and in three of the female cases habitual bilious attacks were experienced in childhood and for many years afterwards.

The nature of this distant influence of gout is unexplained. It may tell in two ways: First, by the inherited delicacy and irritability of the nervous system, analogous to the irritable skin found in other sufferers; and, in the second place, by perverting the juices.

*Treatment.*—A new method of treatment for this almost incurable disease is described by C. L. Dana.[15] Tic Douloureux, a form of neuralgia of the trigeminal nerve, is destructive in its origin and course. It begins during or near the degenerative period of life, its pains are sharp and agonising, and drive through the face like a red-hot blade, they come on intermittently in crises of intolerable distress, with sweating, lacrymation, nasal discharge, and flushing and puffing of the face. Spontaneous cure not very rarely occurs, and the natural duration of the malady in its milder forms is from six to twelve years.

In many cases it shows no abatement, but lasts till the patient is worn out by the agony. The treatment heretofore advised has been of the most various kinds, and includes nearly every narcotic in the Pharmacopœia, besides tonics, counter-irritants, electricity, hydrotherapy, and surgical intervention. Single cases of cures have been recorded from the use of various remedies (iron, aconitia, glonoin, salicylates, iodides, gelseminum, &c.), but no series of cases treated medicinally with success appear to have been recorded. Dana's personal experience leads him to think that surgical interference usually gives only temporary relief.

The method which he has adopted[16] for two years in hospital and in private practice with satisfactory results, consists of the following procedures: (1) The hypodermic injection of massive doses of strychnine; (2) the administration of stimulants, such as iodide of potassium, and of tonics, including especially large doses of tincture of iron; (3) rest in bed, with light diet and diuretics. The treatment admits of no half-way measures. The patient must take the full course, and sometimes even a second or third course. (4) The use of strychnine. This is given in single daily doses, hypodermically. He usually begins with 1-30th gr., and this is very slowly increased until by the fifteenth or twentieth day 1-6th to 1-4th grain is given. Most patients cannot take over 1-5th gr., an excess being shown by stiffness in the jaws and legs, trembling, and nervousness. Sometimes the largest doses are not well borne, and are not advisable; but this is rare. He has noticed that the large doses, 1-15th to 1-5th gr., often have a decidedly anodyne effect, quieting the patient for hours like a dose of morphine. Sometimes the largest doses temporarily increase the pain, but this is certainly rare. The best results are in those who feel the anodyne effect. After reaching the maximum dose the strychnine should be continued for a week or ten days, and then gradually reduced, so that by the end of five or six weeks the dose commenced with is reached. The drug is then stopped, and the patient is placed on iodide of potassium 5 gr. t. d., increased to 20 gr., and tincture of iron 5 m., increased, if possible to 20 m., and well diluted. In some cases salicylate of potassium replaces the iodide, or nitro-glycerine is added to the iodide of iron. Rest in bed is a point in the management of tic which cannot be too stringently insisted upon. The room should be kept as nearly as possible at 68 deg. Fahr., and the air should be moderately humid. At the end of four weeks the patient is allowed to go out for two hours daily, and at the end of six weeks he can resume his ordinary avocations. Details are given of eight cases (six men, two women) treated in this way; the ages ranged from forty-three to seventy. The branches of the trigeminal nerve most affected were in six of the cases the second, or the second and one other branch. The duration of the malady before the line of treatment described was adopted ranged from two to ten years, averaging six years. Relief was obtained in all but two of the cases, and in one of these the method was not fully tried. The duration of the relief in five cases has been from one year and a half to two years. In two cases treatment was finished four months ago. In three cases there was a slight albuminuria. In one case the disease was complicated with a motor tic, and in one case it was complicated with severe heart lesion, as well as albuminuria. The maximum dose of strychnine in all cases reach 1-5th to 1-4th gr. The author concludes, after much disheartening experience with aconite, iodide, gelseminum, salicylates, tonics of various kinds, electricity, &c., that the method of treatment above described is the most useful.

[1] Amer. Journ. of Med. Science, June, 1896. [2] Senator, Wiener Med. Wochenscrift, Nov. 31, 1894. [3] Kahler and Pick, Vierteljahrschr. f. pract. heilk., 1879; Kojewnikoff, Archiv. de Neurol, vol. vi., 1883; Charcot and Marie, Archiv. de Neurol, July, 1883; Lombroso, Lo Sperimentale, 1888; Lenalem, Archiv. de Neurol, vol. viii., 1887; Mott, Brain, Spring, 1895. [4] Traite de Med., Vol. vi., p. 340. [5] Archiv. f. Psych., xxiv., p. 758. [6] Archiv. de Neurol, Vol x., 1885. [7] Archiv. f. Psych., Vol. x., 1880. [8] Kronthral, Neurolog. Centralb., p. 130, 1891. [9] Oppenheim, Archiv. f. Psych, Vol. xxiv., p. 758. [10] Wolf, Zeitschrift f. Klin. Med., Vol. xxv. [11] R. Schultze, Archiv. f. Klin. Med., Vol. xxiii. [12] Gaz. degli Hospet., Sept. 3, 1895; Journ. of Nerv. and Mental Dis. [13] Medical Week, July 17, 1896. [14] B.M.J., Nov. 21, 1896. [15] The Post Graduate, July, 1896. [16] Ibid., and Chicago Medical Recorder, Aug., 1896.

